# Burnout and Well-Being Among Medical Professionals in China: A National Cross-Sectional Study

**DOI:** 10.3389/fpubh.2021.761706

**Published:** 2022-01-17

**Authors:** Ying Xiao, Dong Dong, Huanyu Zhang, Peipei Chen, Xiangyan Li, Zhuang Tian, Zhicheng Jing, Shuyang Zhang

**Affiliations:** ^1^State Key Laboratory of Complex Severe and Rare Diseases, Department of Cardiology, Peking Union Medical College Hospital, Chinese Academy of Medical Sciences and Peking Union Medical College, Beijing, China; ^2^Shenzhen Research Institute, The Chinese University of Hong Kong, Shenzhen, China; ^3^Faculty of Medicine, JC School of Public Health and Primary Care, The Chinese University of Hong Kong, Shatin, Hong Kong SAR, China; ^4^Medical Science Research Center, Peking Union Medical College Hospital, Chinese Academy of Medical Sciences and Peking Union Medical College, Beijing, China

**Keywords:** burnout, capability well-being, medical professionals, China, national-level

## Abstract

**Objective:**

To determine the profile of Chinese medical professionals with burnout symptoms at the national level and identify the association between capability well-being and burnout.

**Design and Setting:**

A cross-sectional study in a nonrandom national sample of medical staff from 6 provinces across western, central and eastern China.

**Participants:**

Physicians, medical laboratory scientists, nurses, and general practitioners aged 18 years or above who submitted a completed online questionnaire from June 2019 to January 2020 successfully (*N* = 25,120).

**Main Outcome Measures:**

The prevalence of burnout symptoms was assessed by the 22-item Maslach Burnout Inventory-Human Services Survey (MBI-HSS), which consists of three domains: emotional exhaustion (EE), depersonalization (DP), and personal achievement (PA). The overall high burnout was defined as EE score ≥27 or DP score ≥10. The capability well-being was measured by the Investigating Choice Experiments Capability Measure for Adults (ICECAP-A) and the overall ICECAP-A score was calculated using the UK value set, ranging from a score of 0–1. Multivariable logistic regression analysis was used to identify the association between well-being and the overall high burnout.

**Results:**

Among the 25,120 participants, 60.8% of the participants reported at least one symptom of burnout, whereas 11.2% reported all three symptoms of burnout. In the adjusted model, ICECAP-A score was independently associated with high burnout (AOR = 0.018, 95% CI = 0.015–0.022). Medical staff who were males, with shorter working years, working in tertiary hospitals, and those with the specialties of psychiatry, intensive care, emergency medicine, internal medicine, oncology, and pediatrics were at higher risk of reporting burnout symptoms.

**Conclusion:**

The burnout symptoms were relatively common among Chinese medical staff and they were found to be independently associated with capability well-being in health professionals. Interventions should be enhanced on vulnerable groups to reduce burnout and promote well-being in future studies.

## Introduction

Physician burn-out is a global crisis (1), but Chinese doctors may have suffered more. The ratio of doctors to the general population in China is 1:735, which is substantially lower than that in Western countries (where the ratio ranges from 1:280 to 1:640) (2). Yet the violence against the doctors is much higher (3). The number of medical staff injured during medical disputes rose from 2,604 in 2002 to 5,519 in 2006 and to 17,000 in 2010 (4, 5). According to the 2016 and 2017 surveys by the Chinese Medical Doctor Association (2018), more than 60% of medical workers surveyed experienced doctor–patient conflicts (6). More than 63 percent of all hospitals across the country have had their personnel injured, disabled, or even killed by disgruntled patients and their relatives (7). It is therefore not surprising that Chinese medical staff has suffered from a large variety of physical and mental health problems, including burnout (8).

Burnout was newly included the 11th Revision of International Classification of Diseases (ICD-11) in May 2019 as a multi-dimensional syndrome consisting of emotional exhaustion, depersonalization, and diminished feelings of personal accomplishment (9). Burnout and occupational stress among Chinese medical professionals has attracted international attention nowadays (10). Previous studies (11) showed that the prevalence of burnout symptoms among Chinese doctors ranged from 66.5% to 87.8%. However, few study has been conducted to assess the prevalence of burnout among Chinese medical professionals at the national level (12). Unlike depression or occupational fatigue, people with burnout are exposed to high levels of work-related stress (13, 14). Previous studies (15–18) have shown that among medical staff, burnout has adverse effects on health conditions and overall well-being, which could lead to depressive symptoms and suicidal ideations. Moreover, burnout has been associated with job dissatisfaction, frequent job turnover, and increased medical malpractice or errors (19, 20). Hence, burnout is an underlying barrier to the well-being of medical staff and the quality of healthcare.

Traditionally, well-being is a health-related measurement of quality of life comprising physiological, psychological and behavioral dimensions (21). A recent systematic review (15) synthesized 19 studies and found that job burnout could cause negative impact on well-being among human service workers including healthcare providers. However, health-related well-being may not be able to capture multi-dimensional outcomes such as interests of carers, family or society (22). Under the circumstances, the Sen's (23) capability approach has been developed to provide more comprehensive measures of well-being, one of which is the Investigating Choice Experiments Capability Measure for Adults (ICECAP-A). ICECAP-A is a general instrument developed in the UK to measure capability well-being (24). It is intended for measuring a person's ability to achieve important ‘functioning’, which consists of five attributes: stability, attachment, autonomy, achievement and enjoyment (19). So far, to our best knowledge, no studies have been performed to examine the relationship between burnout and capability well-being among medical staff in China or in other countries. The aim of this study was to explore the profile of Chinese medical professionals with burnout syndromes at the national level, and to identify the association between capability well-being and burnout among Chinese medical staff.

## Methods

### Study Design and Participants

This is a cross-sectional study at the national level performed by the Peking Union Medical College Hospital (PUMCH) and the Chinese University of Hong Kong (CUHK) from June 2019 to January 2020. The study was conducted across the hospitals in both urban and rural health care systems in China. To improve the sample representativeness, 6 of the 31 provincial-level regions in mainland China were surveyed, namely, Shandong, Shanxi, Shaanxi, Jiangsu, Jiangxi, and Sichuan. These selected provinces represented diverse geographical locations (eastern, central, and western China), socioeconomic status (high, middle, and low gross domestic product per capita), and number of health technical staff per 1,000 persons.

The Institutional Review Board of Peking Union Medical College Hospital approved this study. Physicians, medical laboratory scientists, nurses and general practitioners aged 18 years or above from around 400 hospitals in the six provinces were invited to participate in this project. The questionnaire was uploaded to an online survey platform named Wen Juan Xing. Link to the questionnaire was distributed along with the invitation letter. Although the survey mainly involved a non-random convenience sampling method, the total number of participants accounts for 1.3% of all medical professionals from the six provinces, which makes the sample size considerably large enough. All the participants were anonymized and de-identified. The participants must click the “consent to participate” button at the beginning of the survey. Otherwise, the survey would be stopped immediately, and the participants would be taken away from the questionnaire. The exclusion criteria for the valid responses were as follows: total completion time <420 s (420 s generally represented the values for completing questionnaire at or above the 95th-percentile time of general population), selection of the same options throughout the survey.

### Study Measures

The demographic characteristics of Chinese medical staff were investigated in the questionnaire including gender, age, birthplace, monthly income, education, working title, working years, hospital class, and specialty. The participants were also asked to rate on the level of 1–4 (1 = full capability, 4 = no capability) for the measurement of well-being using the ICECAP-A. The overall ICECAP score was calculated using the UK value set, ranging from a score of 0–1 (22). The use of the Chinese version of ICECAP-A has obtained permission from the ICECAP team at the Institute of Applied Health Research at the University of Birmingham.

The 22-item Maslach Burnout Inventory-Human Services Survey (MBI-HSS) was used to measure burnout symptoms in this study (25), which encompasses three domains with corresponding subscales: emotional exhaustion (EE), depersonalization (DP), and personal achievement (PA). Participants were asked to respond using a seven-point Likert scale ranging from a score of 0–6 (0 = never, 6 = everyday). According to previous literature (26), the high score in each domain was designated as follows: EE score ≥27, DP score ≥10, and PA score ≤33. The overall high burnout was defined as: EE score ≥27 or DP score ≥10 (27). Cronbach's alpha coefficient was used for the reliability analysis of the MBI-HSS, with a value >0.7 indicating a high level of internal consistency (28).

### Statistical Analysis

Frequencies, percentages, means, and standard deviations were analyzed for descriptive data based on regional locations (i.e., eastern, central, and western China). Univariable analysis was performed to evaluate the association between the characteristics of Chinese medical professionals and burnout symptoms. Independent sample *t*-tests were used for continuous variables to compare differences, and Fisher's exact tests or chi-square testes were used for categorical variables as appropriate. In the multivariable regression analysis, adjusted odds ratios (AOR) were reported with 95% confidence intervals and a *p* < 0.05 was considered statistically significant. The variance inflation factor (VIF) for each independent variable in the multivariable regression model was examined to eliminate collinearity. Statistical analysis was performed using SPSS, version 25.0 (Armonk, NY, USA: IBM; 2019) by two independent researchers.

## Results

Of the 53,636 eligible health professionals who opened the web link, 28,745 (53.6%) completed the survey. After the exclusion of missing and invalid data, 25,120 (87.4%) participants were ultimately included in the study. Among them, 73.7% were females, 51.3% were aged 26–35 years, and 87.2% reported a bachelor or higher degree. The majority (88.5%) of the participants work in tertiary hospitals and more than half (58.9%) of them have worked <10 years. Table 1 shows more detailed information on the demographic characteristics of the study sample.

**Table 1:** Demographic characteristics of medical professionals in China.

**Characteristics**	**Total** ***n* = 25,120**	**Eastern** ***n* = 8,047**	**Central** ***n* = 7,639**	**Western** ***n* = 9,434**
	**Number (percent)**			
**Gender**				
Male	6,613 (26.3)	2,081 (25.9)	1,909 (25.0)	2,623 (27.8)
Female	18,507 (73.7)	5,966 (74.1)	5,730 (75.0)	6,811 (72.2)
**Age**				
18–25	2,406 (9.6)	635 (7.9)	555 (7.3)	1,216 (12.9)
26–35	12,874 (51.3)	4,207 (52.3)	3,803 (49.8)	4,864 (51.6)
36–45	6,369 (25.4)	2,093 (26.0)	2,006 (26.3)	2,270 (24.1)
46–55	2,951 (11.7)	961 (11.9)	1,066 (14.0)	924 (9.8)
56–65	483 (1.9)	142 (1.8)	196 (2.6)	145 (1.5)
66+	37 (0.1)	9 (0.1)	13 (0.2)	15 (0.2)
**Birthplace**				
Urban area	19,043 (75.8)	6,193 (77.0)	6,360 (83.3)	6,490 (68.8)
Rural area	6,044 (24.1)	1,844 (22.9)	1,269 (16.6)	2,931 (31.1)
Others	33 (0.1)	10 (0.1)	10 (0.1)	13 (0.1)
**Monthly income**				
<5,000	8,922 (35.5)	1,221 (15.2)	3,506 (45.9)	4,195 (44.5)
5,000–10,000	14,196 (56.5)	5,709 (70.9)	3,867 (50.6)	4,620 (49.0)
10,000–30,000	1,874 (7.5)	1,095 (13.6)	227 (3.0)	552 (5.9)
30,000–50,000	43 (0.2)	10 (0.1)	11 (0.1)	22 (0.2)
>50,000	85 (0.3)	12 (0.1)	28 (0.4)	45 (0.5)
**Province**				
Shandong	3,005 (12.0)	3,005 (37.3)	0 (0.0)	0 (0.0)
Jiangsu	5,042 (20.1)	5,042 (62.7)	0 (0.0)	0 (0.0)
Jiangxi	2,838 (11.3)	0 (0.0)	2,838 (37.2)	0 (0.0)
Shanxi	4,801 (19.1)	0 (0.0)	4,801 (62.8)	0 (0.0)
Sichuan	1,824 (7.3)	0 (0.0)	0 (0.0)	1,824 (19.3)
Shanxi	7,610 (30.3)	0 (0.0)	0 (0.0)	7,610 (80.7)
**Education**				
Secondary vocational school	209 (0.8)	32 (0.4)	35 (0.5)	142 (1.5)
Three-year college	3,020 (12.0)	440 (5.5)	774 (10.1)	1,806 (19.1)
Bachelor's degree	15,134 (60.2)	4,629 (57.5)	5,012 (65.6)	5,493 (58.2)
Master's degree	5,898 (23.5)	2,481 (30.8)	1,748 (22.9)	1,669 (17.7)
Doctorate/postdoc	859 (3.4)	465 (5.8)	70 (0.9)	324 (3.4)
**Title**				
Primary	11,307 (45.0)	3,028 (37.6)	3,297 (43.2)	4,982 (52.8)
Middle	8,563 (34.1)	3,123 (38.8)	2,741 (35.9)	2,699 (28.6)
Vice-senior	3,310 (13.2)	1,205 (15.0)	1,035 (13.5)	1,070 (11.3)
Senior	1,316 (5.2)	571 (7.1)	433 (5.7)	312 (3.3)
None	624 (2.5)	120 (1.5)	133 (1.7)	371 (3.9)
**Working years**				
≤ 5	7,627 (30.4)	2,212 (27.5)	1,897 (24.8)	3,518 (37.3)
6–10	7,155 (28.5)	2,422 (30.1)	2,222 (29.1)	2,511 (26.6)
11–15	3,932 (15.7)	1,226 (15.2)	1,385 (18.1)	1,321 (14.0)
16–20	2,113 (8.4)	736 (9.1)	618 (8.1)	759 (8.0)
21–25	1,887 (7.5)	648 (8.1)	612 (8.0)	627 (6.6)
26–30	1,441 (5.7)	490 (6.1)	517 (6.8)	434 (4.6)
30+	965 (3.8)	313 (3.9)	388 (5.1)	264 (2.8)
**Hospital class**				
Tertiary hospital	22,220 (88.5)	8,039 (99.9)	7,625 (99.8)	6,226 (66.0)
Secondary hospital	3,081 (12.3)	6 (0.1)	8 (0.1)	3,001 (31.8)
Primary hospital	221 (0.9)	2 (0.0)	6 (0.1)	207 (2.2)
**Specialty**				
Anesthesiology	588 (2.3)	129 (1.6)	146 (1.9)	313 (3.3)
Dermatology	324 (1.3)	119 (1.5)	111 (1.5)	94 (1.0)
Emergency medicine	797 (3.2)	207 (2.6)	312 (4.1)	278 (2.9)
Infectious diseases	796 (3.2)	161 (2.0)	271 (3.5)	364 (3.9)
Intensive care	928 (3.7)	407 (5.1)	327 (4.3)	194 (2.1)
Internal medicine	6,983 (27.8)	2,781 (34.6)	2,043 (26.7)	2,159 (22.9)
Laboratory medicine	720 (2.9)	53 (0.7)	192 (2.5)	475 (5.0)
Obstetrics and gynecology	1,670 (6.6)	472 (5.9)	406 (5.3)	792 (8.4)
Oncology	578 (2.3)	257 (3.2)	188 (2.5)	133 (1.4)
Ophthalmology	464 (1.8)	116 (1.4)	211 (2.8)	137 (1.5)
Orthopedic surgery, medical cosmetology	89 (0.4)	22 (0.3)	30 (0.4)	37 (0.4)
Otolaryngology	327 (1.3)	118 (1.5)	92 (1.2)	117 (1.2)
Pain medicine	118 (0.5)	26 (0.3)	31 (0.4)	61 (0.6)
Pathology	87 (0.3)	10 (0.1)	40 (0.5)	37 (0.4)
Pediatrics	1,742 (6.9)	534 (6.6)	682 (8.9)	526 (5.6)
Psychiatry	255 (1.0)	53 (0.7)	8 (0.1)	194 (2.1)
Radiology	1,363 (5.4)	261 (3.2)	282 (3.7)	820 (8.7)
Sports medicine, rehabilitation	729 (2.9)	182 (2.3)	187 (2.4)	360 (3.8)
Stomatology	337 (1.3)	86 (1.1)	110 (1.4)	141 (1.5)
Surgery	3,757 (15.0)	1,653 (20.5)	1,186 (15.5)	918 (9.7)
Traditional Chinese medicine	491 (2.0)	64 (0.8)	154 (2.0)	273 (2.9)
Others	1,977 (7.9)	336 (4.2)	630 (8.2)	1,011 (10.7)

The prevalence rates of burnout symptoms among Chinese medical staff are shown in Table 2. Among the 25,120 participants, 35.5% of them experienced high EE, 30.0% experienced high DP, and 38.4% had a low sense of PA. Overall, 60.8% of the participants reported at least one symptom of burnout, whereas 11.2% reported all three symptoms of burnout. The Cronbach's alpha coefficients for EE, DP, and PA subscales were 0.891, 0.812, and 0.866, respectively, indicating a high level of reliability. Table 3 shows the frequencies and percentages of the participants responding to ICECAP-A. For all attributes, the second-best level was the most commonly selected option, ranging from 43.5% for achievement to 63.4% for enjoyment.

**Table 2:** Measures of burnout among medical professionals in China.

**MBI-HSS scale**	**Total**	**Eastern**	**Central**	**Western**
**EE subscale, range 0–54**				
Mean score (SD)	23 (11.6)	23.5 (11.4)	23 (11.8)	22.7 (11.5)
Score 0–26^b^ (%)	16,213 (64.5)	5,069 (63.0)	4,935 (64.6)	6,209 (65.8)
Score 27–54^a^ (%)	8,907 (35.5)	2,978 (37.0)	2,704 (35.4)	3,225 (34.2)
**DP subscale, range 0–30**				
Mean score (SD)	7.1 (6.0)	7.5 (6.1)	7.0 (6.0)	6.9 (5.9)
Score 0–9^b^ (%)	17,583 (70.0)	5,427 (67.4)	5,392 (70.6)	6,764 (71.7)
Score 10–30^a^ (%)	7,537 (30.0)	2,620 (32.6)	2,247 (29.4)	2,670 (28.3)
**PA subscale, range 0–48**				
Mean score (SD)	34.8 (9.6)	35.6 (9.1)	34.4 (9.9)	34.6 (9.7)
Score 34–48^b^ (%)	15,474 (61.6)	5,216 (64.8)	4,509 (59.0)	5,749 (60.9)
Score 0–33^a^ (%)	9,646 (38.4)	2,831 (35.2)	3,130 (41.0)	3,685 (39.1)
**Overall high burnout^c^**				
EE/DP/PA (%)	15,285 (60.8)	4,800 (59.6)	4,768 (62.4)	5,717 (60.6)
EE & DP& PA (%)	2,802 (11.2)	957 (11.9)	872 (11.4)	973 (10.3)
EE/DP (%)	11,110 (44.2)	3,723 (46.3)	3,373 (44.2)	4,014 (42.5)

*MBI-HSS, Maslach Burnout Inventory-Human Services Survey; EE, Emotional exhaustion; DP, Depersonalization; PA, Personal accomplishment; SD, Standard deviation*.

a *High burnout*.

b *Low or moderate burnout*.

c *Three frequently used definitions were employed to determine overall high burnout*.

**Table 3:** Responses to the ICECAP-A scale among Chinese medical professionals.

**Attributes**	**Overall**	**Eastern**	**Central**	**Western**
	**Number (percent)**			
**Stability**				
4^a^	5,270 (21.0)	1,582 (19.7)	1,703 (22.3)	1,985 (21.0)
3	13,842 (55.1)	4,834 (60.1)	4,030 (52.8)	4,978 (52.8)
2	5,306 (21.1)	1,478 (18.4)	1,653 (21.6)	2,175 (23.1)
1	702 (2.8)	153 (1.9)	253 (3.3)	296 (3.1)
**Attachment**				
4	6,254 (24.9)	2,093 (26.0)	1,952 (25.6)	2,209 (23.4)
3	15,401 (61.3)	5,059 (62.9)	4,547 (59.5)	5,795 (61.4)
2	3,304 (13.2)	857 (10.6)	1,077 (14.1)	1,370 (14.5)
1	161 (0.6)	38 (0.5)	63 (0.8)	60 (0.6)
**Autonomy**				
4	7,391 (29.4)	2,377 (29.5)	2,242 (29.3)	2,772 (29.4)
3	13,967 (55.6)	4,701 (58.4)	4,141 (54.2)	5,125 (54.3)
2	3,684 (14.7)	947 (11.8)	1,226 (16.0)	1,511 (16.0)
1	78 (0.3)	22 (0.3)	30 (0.4)	26 (0.3)
**Achievement**				
4	4,111 (16.4)	1,208 (15.0)	1,383 (18.1)	1,520 (16.1)
3	10,917 (43.5)	3,699 (46.0)	3,252 (42.6)	3,966 (42.0)
2	9,856 (39.2)	3,075 (38.2)	2,927 (38.3)	3,854 (40.9)
1	236 (0.9)	65 (0.8)	77 (1.0)	94 (1.0)
**Enjoyment**				
4	6,446 (25.7)	2,067 (25.7)	1,996 (26.1)	2,383 (25.3)
3	15,931 (63.4)	5,194 (64.5)	4,727 (61.9)	6,010 (63.7)
2	2,564 (10.2)	743 (9.2)	853 (11.2)	968 (10.3)
1	179 (0.7)	43 (0.5)	63 (0.8)	73 (0.8)

a *Refers to the capacity score at each attribute level*.

The characteristics of Chinese medical professionals with or without an overall high burnout were compared in Table 4. Of the 11,110 (44.2%) participants with an overall high burnout, the average ICECAP-A score (mean ± SD) was 0.7568 ± 0.1622, which was lower than those of participants without an overall high burnout (0.8318 ± 0.1154). Significant differences between participants with or without an overall high burnout were found in gender, age, location, education, working title, working years, hospital class, specialty and ICECAP-A score (*p* < 0.001); these variables were further included in the multivariable logistic regression model. However, the variables of age and working years were found to have a value of VIF larger than 10, which indicated the occurrence of collinearity. Therefore, we only included the variable of working years instead of both variables in the multivariable regression model. In the final reported model, all the variables were found to have a value of VIF below 5, which indicated no problem of collinearity.

**Table 4:** Characteristics of Chinese medical professionals associated with high burnout^a^.

**Characteristics**	**High burnout** ***n* = 11,110**	**Low/moderate burnout** ***n* = 14,010**	***p*-value^c^**
	**Number (percent)**		
**Gender**			**<0.001**
Male	3,177 (28.6)	3,436 (24.5)	
Female	7,933 (71.4)	10,574 (75.5)	
**Age**			**<0.001**
18–25	1,062 (9.6)	1,344 (9.6)	
26–35	5,967 (53.7)	6,907 (49.3)	
36–45	2,824 (25.4)	3,545 (25.3)	
46–55	1,081 (9.7)	1,870 (13.3)	
56–65	158 (1.4)	325 (2.3)	
66+	18 (0.2)	19 (0.1)	
**Birthplace**			0.938
Urban area	8,413 (75.7)	10,630 (75.9)	
Rural area	2,683 (24.1)	3,361 (24.0)	
Others	14 (0.1)	19 (0.1)	
**Monthly income**			0.089
<5,000	4,022 (36.2)	4,900 (35.0)	
5,000–10,000	6,246 (56.2)	7,950 (56.7)	
10,000–30,000	794 (7.1)	1,080 (7.7)	
30,000–50,000	15 (0.1)	28 (0.2)	
>50,000	33 (0.3)	52 (0.4)	
**Location**			**<0.001**
Eastern	3,723 (33.5)	4,324 (30.9)	
Central	3,373 (30.4)	4,266 (30.4)	
Western	4,014 (36.1)	5,420 (38.7)	
**Education**			**<0.001**
Secondary vocational school	69 (0.6)	140 (1.0)	
Three-year college	1,262 (11.4)	1,758 (12.5)	
Bachelor's degree	6,555 (59.0)	8,579 (61.2)	
Master's degree	2,801 (25.2)	3,097 (22.1)	
Doctorate/postdoc	423 (3.8)	436 (3.1)	
**Title**			**<0.001**
Primary	5,110 (46.0)	6,197 (44.2)	
Middle	3,928 (35.4)	4,635 (33.1)	
Vice-senior	1,343 (12.1)	1,967 (14.0)	
Senior	472 (4.2)	844 (6.0)	
None	257 (2.3)	367 (2.6)	
**Working years**			**<0.001**
≤ 5	3,490 (31.4)	4,137 (29.5)	
6–10	3,358 (30.2)	3,797 (27.1)	
11–15	1,838 (16.5)	2,094 (14.9)	
16–20	902 (8.1)	1,211 (8.6)	
21–25	712 (6.4)	1,175 (8.4)	
26–30	490 (4.4)	951 (6.8)	
30+	320 (2.9)	645 (4.6)	
**Hospital class**			**<0.001**
Tertiary hospital	9,757 (87.8)	12,133 (86.6)	
Secondary hospital	1,294 (11.6)	1,721 (12.3)	
Primary hospital	59 (0.5)	156 (1.1)	
**Specialty**			**<0.001**
Anesthesiology	245 (2.2)	343 (2.4)	
Dermatology	123 (1.1)	201 (1.4)	
Emergency medicine	406 (3.7)	391 (2.8)	
Infectious diseases	310 (2.8)	486 (3.5)	
Intensive care	466 (4.2)	462 (3.3)	
Internal medicine	3,409 (30.7)	3,574 (25.5)	
Laboratory medicine	207 (1.9)	513 (3.7)	
Obstetrics and gynecology	682 (6.1)	988 (7.1)	
Oncology	282 (2.5)	296 (2.1)	
Ophthalmology	177 (1.6)	287 (2.0)	
Orthopedic surgery, medical cosmetology	30 (0.3)	59 (0.4)	
Otolaryngology	141 (1.3)	186 (1.3)	
Pain medicine	45 (0.4)	73 (0.5)	
Pathology	25 (0.2)	62 (0.4)	
Pediatrics	804 (7.2)	938 (6.7)	
Psychiatry	122 (1.1)	133 (0.9)	
Radiology	543 (4.9)	820 (5.9)	
Sports medicine, rehabilitation	272 (2.4)	457 (3.3)	
Stomatology	108 (1.0)	229 (1.6)	
Surgery	1,779 (16.0)	1,978 (14.1)	
Traditional Chinese medicine	182 (1.6)	309 (2.2)	
Others	752 (6.8)	1,225 (8.7)	
**ICECAP-A score^b^ [mean (SD)]**	0.7568 (0.1622)	0.8318 (0.1154)	**<0.001**

a *The definition of high burnout was determined by a MBI-HSS EE score ≥27 or a DP score ≥10*.

b *The overall ICECAP-A score was calculated using the UK value set ranging from 0 to 1*.

c *Fisher's exact test or chi-square test was adopted as appropriate*.

*Bold values indicate statistical significance which is defined as p < 0.05*.

Factors associated with an overall high burnout were identified in the multivariable logistic regression analysis (Table 5). Males were more likely to suffer from high burnout than females (AOR = 0.763, 95% CI = 0.716–0.815 for females vs. males). Working longer than 15 years was significantly associated with lower risk of high burnout (AOR = 0.870, 95% CI = 0.766–0.988 for working years 16–20 vs. ≤ 5). Medical professionals working in tertiary hospitals were at greater risk of reporting high burnout than those working in primary hospitals (AOR = 2.003, 95% CI = 1.456–2.789 for tertiary hospitals vs. primary hospitals). Comparing to the specialty of anesthesiology, the specialty with the highest risk of burnout was psychiatry (AOR = 1.605, 95% CI = 1.175–2.191), followed by intensive care (AOR = 1.514, 95% CI = 1.217–1.886), emergency medicine (AOR = 1.471, 95% CI = 1.174–1.844), internal medicine (AOR = 1.469, 95% CI = 1.228–1.759), oncology (AOR = 1.441, 95% CI = 1.131–1.837), and pediatrics (AOR = 1.317, 95% CI = 1.080–1.607). In the adjusted multivariable regression model, ICECAP-A score was independently associated with high burnout (AOR = 0.018, 95% CI = 0.015–0.022).

**Table 5:** Multivariable logistic regression analysis of predictors of high burnout among Chinese medical professionals^a^.

**Characteristics**	**AOR**	**95% CI**	***p*-value^b^**
**Gender (female: male)**	0.763	0.716–0.815	**<0.001**
**Location (ref: central)**			
Eastern	1.084	1.013–1.160	**0.019**
Western	0.981	0.912–1.055	0.604
**Education (ref: secondary vocational school)**			
Three-year college	1.230	0.895–1.704	0.207
Bachelor's degree	1.270	0.928–1.751	0.139
Master's degree	1.271	0.921–1.768	0.149
Doctorate/postdoc	1.460	1.027–2.089	**0.037**
**Title (ref: primary)**			
Middle	1.038	0.961–1.122	0.340
Vice-senior	0.961	0.849–1.088	0.532
Senior	0.881	0.738–1.050	0.158
None	0.832	0.697–0.991	**0.040**
**Working years (ref: ≤5)**			
6–10	1.019	0.945–1.099	0.624
11–15	1.021	0.925–1.128	0.678
16–20	0.870	0.766–0.988	**0.032**
21–25	0.726	0.630–0.837	**<0.001**
26–30	0.655	0.556–0.771	**<0.001**
>30	0.686	0.567–0.829	**<0.001**
**Hospital class (ref: primary hospital)**			
Secondary hospital	1.897	1.374–2.650	**<0.001**
Tertiary hospital	2.003	1.456–2.789	**<0.001**
**Specialty (ref: anesthesiology)**			
Dermatology	0.953	0.711–1.275	0.748
Emergency medicine	1.471	1.174–1.844	**0.001**
Infectious diseases	0.998	0.794–1.253	0.985
Intensive care	1.514	1.217–1.886	**<0.001**
Internal medicine	1.469	1.228–1.759	**<0.001**
Laboratory medicine	0.622	0.489–0.791	**<0.001**
Obstetrics and gynecology	1.130	0.925–1.382	0.232
Oncology	1.441	1.131–1.837	**0.003**
Ophthalmology	1.011	0.779–1.311	0.935
Orthopedic surgery, medical cosmetology	0.810	0.489–1.316	0.402
Otolaryngology	1.153	0.866–1.534	0.330
Pain medicine	0.866	0.562–1.320	0.506
Pathology	0.604	0.357–0.997	0.054
Pediatrics	1.317	1.080–1.607	**0.007**
Psychiatry	1.605	1.175–2.191	**0.003**
Radiology	1.000	0.814–1.229	1.000
Sports medicine, rehabilitation	0.883	0.700–1.114	0.294
Stomatology	0.743	0.553–0.994	**0.046**
Surgery	1.349	1.122–1.625	**0.002**
Traditional Chinese medicine	0.919	0.710–1.189	0.521
Others	1.030	0.846–1.255	0.770
**ICECAP-A score**	0.018	0.015–0.022	**<0.001**

a *The variables with p-values <0·05 in the univariable analysis were further included in the multivariable logistic regression analysis*.

b *Bold values indicate statistical significance which is defined as p < 0.05*.

## Discussion

Burnout symptoms has been common in health professionals since its recognition in the 1970s (29). Regardless of specialties among physicians and nurses, the rates of burnout symptoms ranged from 25 to 60% in western countries (30–33). Based on the findings in the current study, the prevalence of Chinese medical staff exposed to at least one burnout symptom was 60.8%, which was relatively higher than that reported by western countries. To the best knowledge of us, this is the first large-scale national study assessing burnout symptoms among medical staff across eastern, central and western China, which has increased the representativeness and generalizability of the research findings. The majority of prior studies on the evaluations of burnout focused on a group of health professionals with a specific specialty or occupational setting (34–37). This study has assessed burnout symptoms in health professionals across diverse specialties and occupations, which has contributed to an extended picture of the situation of burnout among medical staff.

In this study, capability well-being was found to be independently associated with the overall high burnout after adjustment for the characteristics of Chinese medical staff. This finding indicated that burnout can not only affect the health-related well-being of medical staff, but also lead to a decline in one's ability to achieve more comprehensive outcomes defined by the ICECAP-A. Although the ICECAP-A is a newly developed instrument for measuring capability well-being in UK, the validity of the Chinese version of ICECAP-A has been proved (38), making it appropriate for measuring the general well-being in China. This study was one of the first attempts to explore the association between capability well-being and burnout, which could benefit social care decision-making for policymakers. Nevertheless, the mechanism on how burnout symptoms can affect capability well-being among medical staff is warranted to be studied in both research and clinical settings.

In this study, males were found to be at higher risk for burnout symptoms than females among Chinese medical staff. However, it appear to be inconsistent worldwide. A multinational cross-sectional study investigating 16 Asian countries/regions showed that gender was not associated with burnout among physicians and nurses (39). Several studies (40, 41) conducted in North America indicated that female physicians were at increased risk of burnout symptoms than males; whereas a systematic review revealed that male students experienced greater emotional exhaustion and depersonalization than females in China (42). A possible interpretation is that men are likely to receive higher societal expectations, promotion stress than women from a culture perspective (43), which may lead to higher job burnout. Work-life integration could be another important impact on gender differences in burnout among medical staff. A national study conducted in the US indicated that female physicians were less satisfied with work-life integration than males (44). Compared with female health professionals in western countries, Chinese female medical staff may obtain more assistance from their parents, including childcare and housekeeping, owing to the distinctions in cultural tradition and family composition. Nonetheless, the satisfaction with work-life integration among Chinese medical staff and its association with burnout need to be further explored. Besides, the COVID-19 pandemic could also impact the level of peritraumatic distress and burnout among medical professionals, which should not be neglected.

Previous studies have shown that heavy workload is associated with increased risk of burnout among physicians and nurses (21), which is consistent with the findings in the current study. In this study, the study revealed the significantly negative correlation between length of employment and prevalence of burnout among Chinese medical staff. It is indicated that medical staff in their early career stage are more vulnerable to burnout symptoms. This is probably because young medical staff serving as trainees or junior positions are generally more overloaded with work (2, 14). We also found that medical staff who work in tertiary hospitals were more likely to report burnout symptoms than those work in primary hospitals. In China, the effectiveness of primary care gatekeeping is limited (45), thus patients are more willing to visit tertiary hospitals to see a doctor as long as it is accessible and affordable. Under the circumstances, the heavy workload of medical staff in tertiary hospitals contributed to the high rates of burnout. Furthermore, the risk of reporting burnout symptoms varied across different clinical specialties according to the results in this study, which mirrored the unique workload characteristics in different occupational settings. The specialties with the increased work burden such as psychiatry, intensive care, emergency medicine, and internal medicine, were at the higher possibility of reporting burnout symptoms. Interventions at both individual and organizational levels should be strengthened on the medical staff with a heavy workload to reduce burnout (46), especially those working with shorter years, in tertiary hospitals, and with specific specialties.

To our knowledge, this is the first national study in China to explore the relationship between burnout and well-being of medical professionals. It finds that males, working in tertiary hospitals, and practicing psychiatry, intensive care, emergency medicine, internal medicine, oncology, and pediatrics were at a higher risk of reporting burnout symptoms; while working longer could decrease the risk of burnout. Moreover, higher burnout was associated with lower well-being. Despite these strengths, our study has some limitations that should be noted. First, the study sample was recruited by convenience sampling methods, resulting in the selection bias of the participants by the inclusion of more young medical staff and those working in tertiary hospitals and primary care facilities. Nevertheless, this large-scale study was performed at the national level with the study cohort selected from 6 representative provinces across eastern, central and western China. Thus, we believe the study sample in the current study is able to represent the general population of Chinese medical staff. Second, the overall ICECAP-A score was calculated based on the UK value set, which may not reflect the real-world value of the Chinese population. Further studies are needed to develop Chinese value sets for measuring capability well-being. Third, our study is lack of distinctions among different occupational settings, which resulted in difficulty comparing the prevalence of burnout between different occupational groups within the study or with other studies at national and global levels. To make a proper comparison, more efforts are required to focus on the distinctions of medical staff by occupations for the analysis of burnout in future studies.

In conclusion, the prevalence rates of burnout symptoms were relatively high among Chinese medical staff. Despite the negative effect on health-related well-being, burnout is also found to be independently associated with capability well-being in health professionals. Interventions should be enhanced on the vulnerable groups of medical staff to reduce burnout, including males, those with shorter working years, working in tertiary hospitals, and specialties with heavy workload.

## Data Availability Statement

The datasets presented in this study can be found in online repositories.

## Ethics Statement

The studies involving human participants were reviewed and approved by the Peking Union Medical College Hospital Ethics Committee approved the study (Ref No.: SK-814). The patients/participants provided their written informed consent to participate in this study.

## Author Contributions

SZ and DD developed the idea. YX, DD, and SZ designed the study. DD, YX, XL, ZT, ZJ, and PC were responsible for data collection. YX, HZ, and DD performed the statistical analysis and drafted the manuscript. SZ revised it. All authors had full access to all of the data in the study, can take responsibility for the integrity of the data and the accuracy of the data analysis, and read and approved the final manuscript.

## Funding

This study was funded by the 13th Five-Year National Science and Technology Major Project for New Drugs (No: 2019ZX09734001), National Key Research and Development Program of China (2016YFC0901500), and the Beijing Natural Science Foundation (7192155). The funding source had no role in the study design, in the collection, analysis, interpretation of data, in the writing of the report, and in the decision to submit the article for publication.

## Conflict of Interest

The authors declare that the research was conducted in the absence of any commercial or financial relationships that could be construed as a potential conflict of interest.

## Publisher's Note

All claims expressed in this article are solely those of the authors and do not necessarily represent those of their affiliated organizations, or those of the publisher, the editors and the reviewers. Any product that may be evaluated in this article, or claim that may be made by its manufacturer, is not guaranteed or endorsed by the publisher.

## Abbreviations

ICECAP-A: Investigating Choice Experiments Capability Measure for Adults
MBI-HSS: Maslach Burnout Inventory-Human Services Survey
EE: emotional exhaustion
DP: depersonalization
PA: personal achievement.
